# Characterization of TB-HIV co infected cases of Udupi district in coastal Karnataka

**DOI:** 10.1186/1471-2334-14-S3-P52

**Published:** 2014-05-27

**Authors:** Parashuram Rao, Kiran Chawla, Vishnu Prasad Shenoy, Chiranjay Mukhopadhyay

**Affiliations:** 1 Department of Microbiology, Kasturba Medical College, Manipal, India

## Background

The rapidly increasing HIV epidemic has fuelled the problem of controlling the spread of tuberculosis. There is a lack of data showing the prevalence of HIVTB co infection from coastal areas of Karnataka. This study was planned to look for the TB and HIV co infection in the Udupi district of Coastal Karnataka and to detect the drug resistance in the study population.

## Methods

750 smear positive TB cases from Udupi district was studied for HIV co infection. The sputum samples from these cases were processed with modified Petroff’s method and cultured on Lowenstein Jensen media. Drug sensitivity testing for *Mycobacterium tuberculosis* was performed by proportion method.

## Results

HIV TB co infection was observed in 6.8% (51/750) of the studied population. Male:female ratio is 7.5:1. Maximum cases 29 (56.8%) were in the age group of 36-50 years. Ratio of cases in Udupi: Kundapura: Karkala Taluks was 18:22:11. 42(82.3%) cases belong to category 1 whereas 9 (17.6%) were of category 2. Multi drug resistance was detected in 3 out of 47 (5.8%) culture positive HIV TB co infected cases, in comparison to 3% of HIV negative but TB positive cases. Mortality of HIV TB co infected cases was observed in 5.8%, as compare to 1.1% in HIV negative but TB positive cases.

## Conclusion

This is the cardinal study highlighting TB-HIV co infection in this coastal area. It shows greater prevalence of MDR in TB-HIV co infected cases thereby stressing the importance of drug sensitivity testing in these patients.

